# Supplementary material for: Spatial-temporal clustering analysis of yaws on Lihir Island, Papua New Guinea to enhance planning and implementation of eradication programs
Source: PLoS Negl Trop Dis. 2018 Oct 29;12(10):e0006840. doi: 10.1371/journal.pntd.0006840 (PMC6224128; doi:10.1371/journal.pntd.0006840)
Supplement: S1 Table — This table lists the spatial-temporal yaws clusters identified by SaTScan using the discrete Poisson method and restricting to the 1187 yaws cases with a confirmed RPR-positive test result. (PDF) [file pntd.0006840.s001.pdf]

S1 Table. Discrete Poisson analysis adjusted for age and sex, confirmed rapid plasma reagin (RPR)-positive. This table lists the spatial-temporal yaws clusters identified by SaTScan using the discrete Poisson method and restricting to the 1187 yaws cases with a confirmed RPR-positive test result.

ID	Start Date	End Date	Number of Villages	Village IDs	Observed Cases	Expected Cases	P-Value
1	2010/8/2	2012/4/29	6	Tumbuapil, Lissel, Komat, Lataul, Kinami, Pangoh	97	21.56	$< 1 \times 10^{-17}$
2	2005/4/11	2009/3/1	4	Putput_1, Putput_2, Lipuko, Matakues	171	83.53	1.0×10^{-11}
3	2014/1/20	2016/5/29	4	Kunaye_1, Kunaye_2, Kul, Zuen	116	52.12	9.4×10^{-9}
