# Supplementary material for: Spatial-temporal clustering analysis of yaws on Lihir Island, Papua New Guinea to enhance planning and implementation of eradication programs
Source: PLoS Negl Trop Dis. 2018 Oct 29;12(10):e0006840. doi: 10.1371/journal.pntd.0006840 (PMC6224128; doi:10.1371/journal.pntd.0006840)
Supplement: S2 Table — This table lists the spatial-temporal yaws clusters identified by SaTScan using the discrete Poisson method but without adjusting for age or sex. (PDF) [file pntd.0006840.s002.pdf]

**S2 Table. Discrete Poisson analysis (unadjusted).** This table lists the spatial-temporal yaws clusters identified by SaTScan using the discrete Poisson method but without adjusting for age or sex.

| ID | Start Date | End Date | Number of Villages | Village IDs | Observed Cases | Expected Cases | P-Value |
|---|---|---|---|---|---|---|---|
| 1 | 2010/8/2 | 2012/3/11 | 6 | Tumbuapil, Lissel, Komat, Lataul, Kinami, Pangoh | 104 | 28.90 | $< 1 \times 10^{-17}$ |
| 2 | 2005/4/11 | 2008/6/8 | 4 | Lipuko, Putput_2, Matakues, Putput_1 | 178 | 79.79 | $1.2 \times 10^{-14}$ |
| 3 | 2009/8/17 | 2014/12/14 | 2 | Kunaye_1, Kunaye_2 | 214 | 119.75 | $2.7 \times 10^{-9}$ |
