# Supplementary material for: Spatial-temporal clustering analysis of yaws on Lihir Island, Papua New Guinea to enhance planning and implementation of eradication programs
Source: PLoS Negl Trop Dis. 2018 Oct 29;12(10):e0006840. doi: 10.1371/journal.pntd.0006840 (PMC6224128; doi:10.1371/journal.pntd.0006840)
Supplement: S3 Table — This table lists the spatial-temporal yaws clusters identified by SaTScan using the space-time permutation method, which uses information about the timing and location of cases but not information about the underlying population at risk. (PDF) [file pntd.0006840.s003.pdf]

S3 Table. Space-time permutation analysis adjusted for age and sex. This table lists the spatial-temporal yaws clusters identified by SaTScan using the space-time permutation method, which uses information about the timing and location of cases but not information about the underlying population at risk.

ID	Start Date	End Date	Number of Villages	Village IDs	Observed Cases	Expected Cases	P-Value
1	2010/8/2	2012/3/11	6	Tumbuapil, Lissel, Komat, Lataul, Kinami, Pangoh	104	44.14	9.0×10^{-13}
2	2005/4/11	2008/6/1	4	Lipuko, Putput_2, Matakues, Putput_1	177	103.80	5.3×10^{-9}
3	2012/5/7	2016/5/29	5	Kul, Kunaye_1, Kunaye_2, Londolovit, Zuen	240	159.64	2.5×10^{-7}
