# Supplementary material for: Spatial-temporal clustering analysis of yaws on Lihir Island, Papua New Guinea to enhance planning and implementation of eradication programs
Source: PLoS Negl Trop Dis. 2018 Oct 29;12(10):e0006840. doi: 10.1371/journal.pntd.0006840 (PMC6224128; doi:10.1371/journal.pntd.0006840)
Supplement: S4 Table — This table lists the spatial-temporal yaws clusters identified by SaTScan using the space-time permutation method but without adjusting for age or sex. (PDF) [file pntd.0006840.s004.pdf]

S4 Table. Space-time permutation analysis (unadjusted). This table lists the spatial-temporal yaws clusters identified by SaTScan using the space-time permutation method but without adjusting for age or sex.

ID	Start Date	End Date	Number of Villages	Village IDs	Observed Cases	Expected Cases	P-Value
1	2010/8/2	2012/3/11	6	Tumbuapil, Lissel, Komat, Lataul, Kinami, Pangoh	104	43.21	3.0×10^{-12}
2	2005/4/11	2008/6/1	4	Lipuko, Putput_2, Matakues, Putput_1	177	102.24	9.4×10^{-9}
3	2012/5/7	2016/5/29	5	Kul, Kunaye_1, Kunaye_2, Londolovit, Zuen	240	157.44	3.0×10^{-7}
