# Supplementary figures and images for: Spatial-temporal clustering analysis of yaws on Lihir Island, Papua New Guinea to enhance planning and implementation of eradication programs
Source: PLoS Negl Trop Dis. 2018 Oct 29;12(10):e0006840. doi: 10.1371/journal.pntd.0006840 (PMC6224128; doi:10.1371/journal.pntd.0006840)

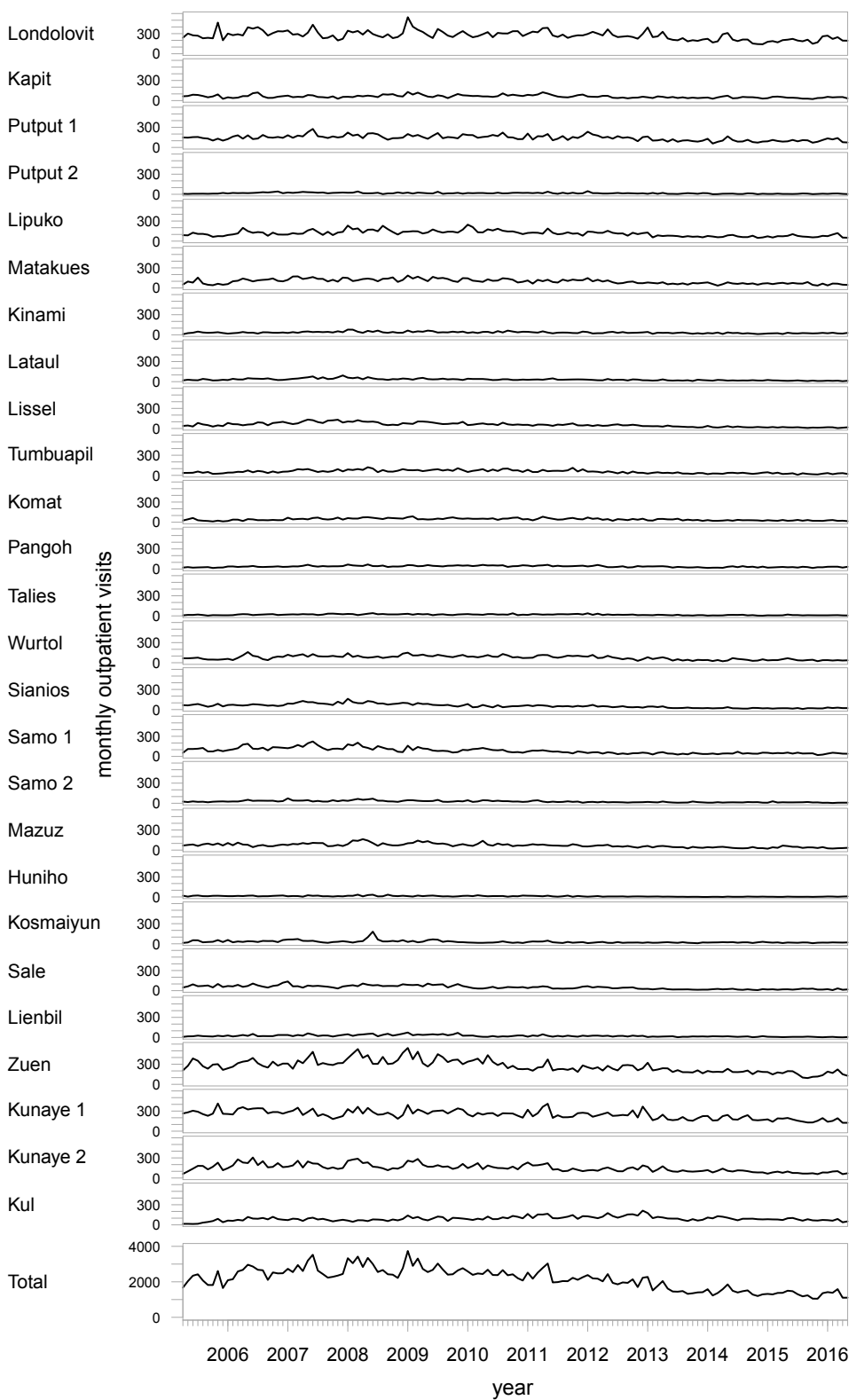

Supplement: S1 Fig — Time series of outpatient visits (any diagnosis) aggregated by month by village and for all villages combined. The villages are ordered to match their sequential order around the circumference of Lihir. (PDF) [file pntd.0006840.s005.pdf]

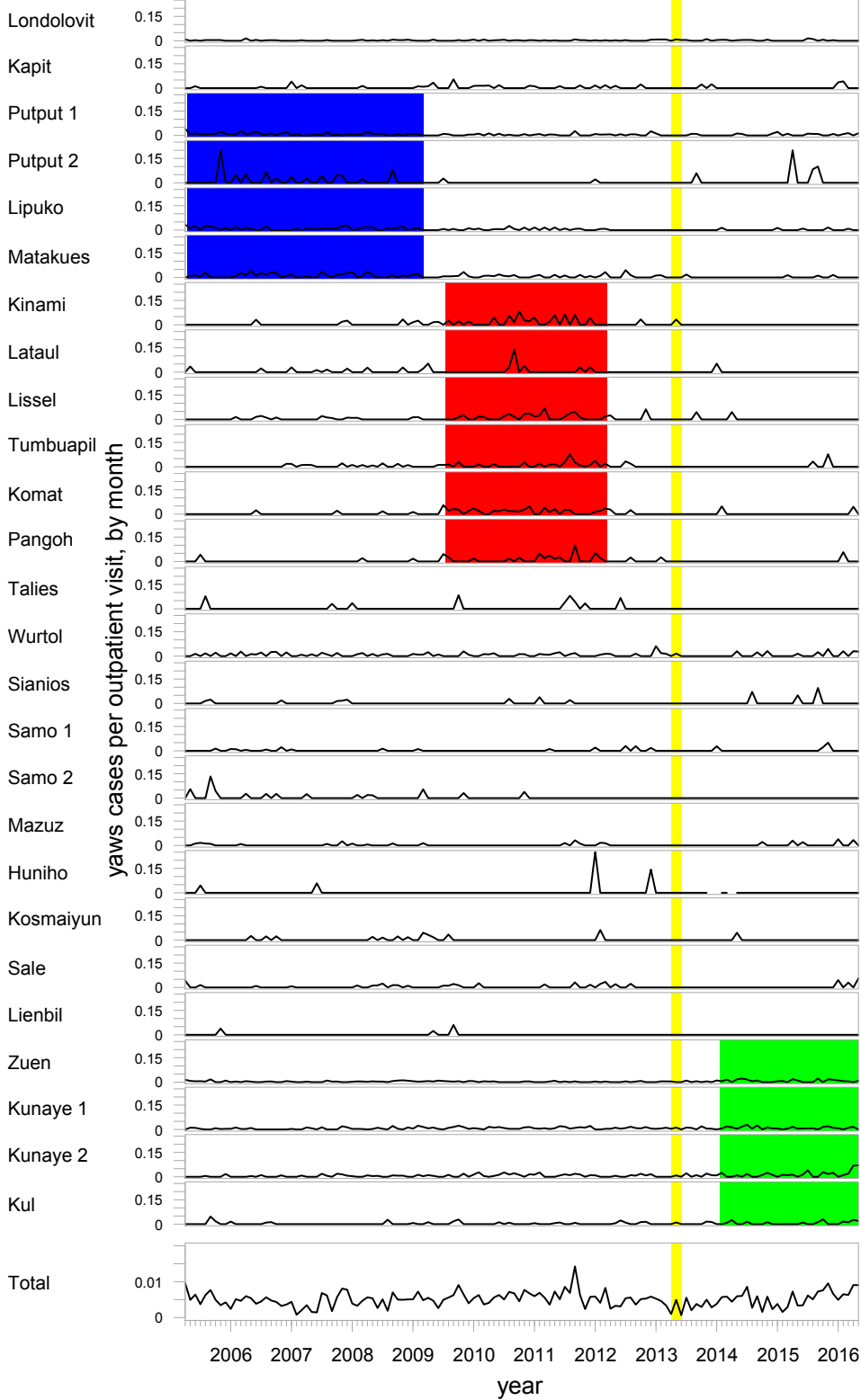

Supplement: S2 Fig — The time series show the proportion of outpatient yaws diagnoses as a fraction of all outpatient visits at the Lihir Medical Centre aggregated by month by village and for all villages combined. The villages are ordered to match their sequential order around the circumference of Lihir. Red, blue, and green rectangles correspond to spatial-temporal clusters 1, 2, and 3, respectively, from Fig 3. The vertical yellow bar corresponds to when mass drug administration was implemented on Lihir. (PDF) [file pntd.0006840.s006.pdf]

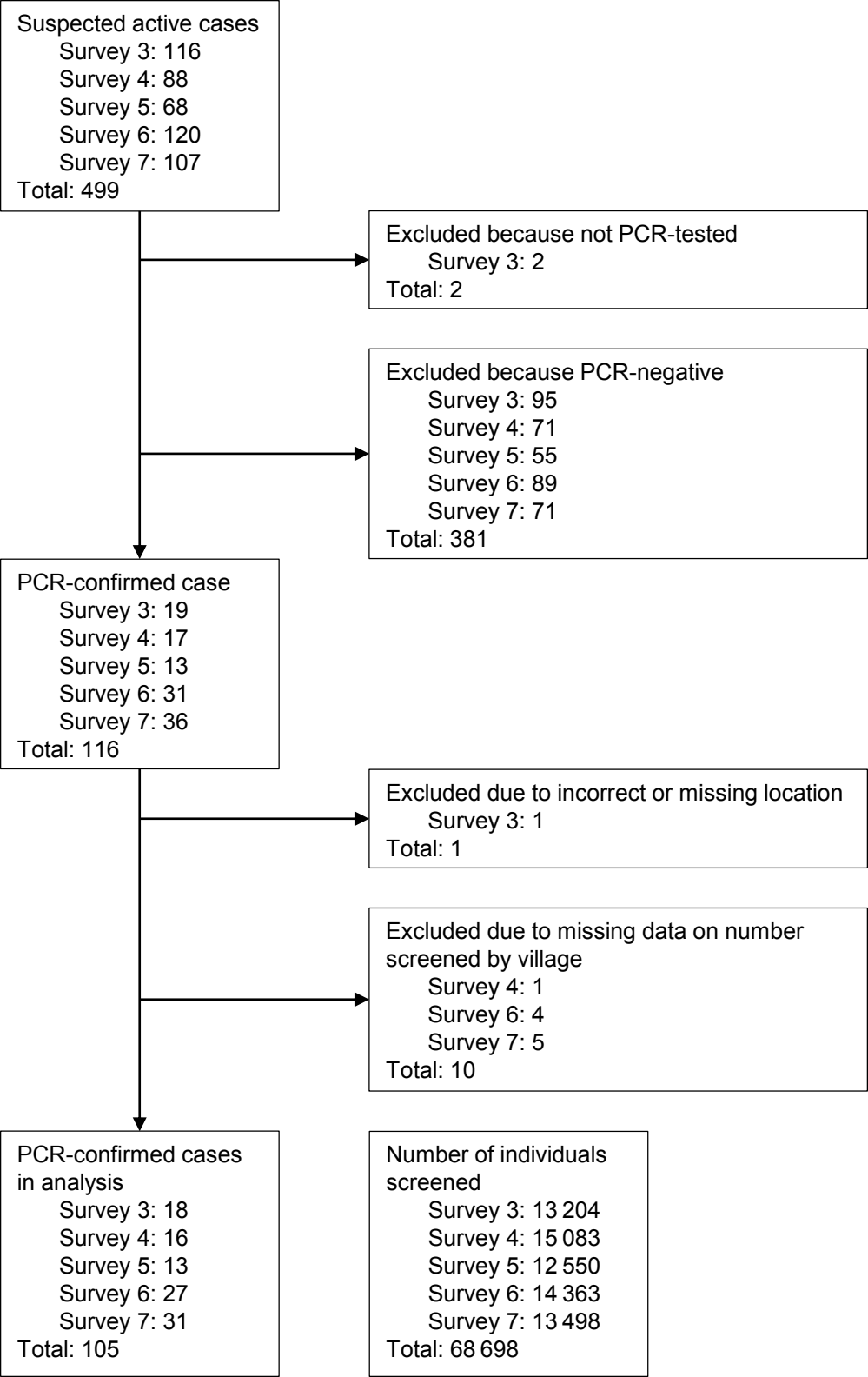

Supplement: S3 Fig — The diagram lists by active case finding survey the number of suspected active cases, PCR-confirmed cases, excluded cases, PCR-confirmed cases in the final analysis, and number of individuals screened. (PDF) [file pntd.0006840.s007.pdf]

| survey | obs. | exp. | obs./exp. | p-value |
|--------|------|------|-----------|---------|
|--------|------|------|-----------|---------|

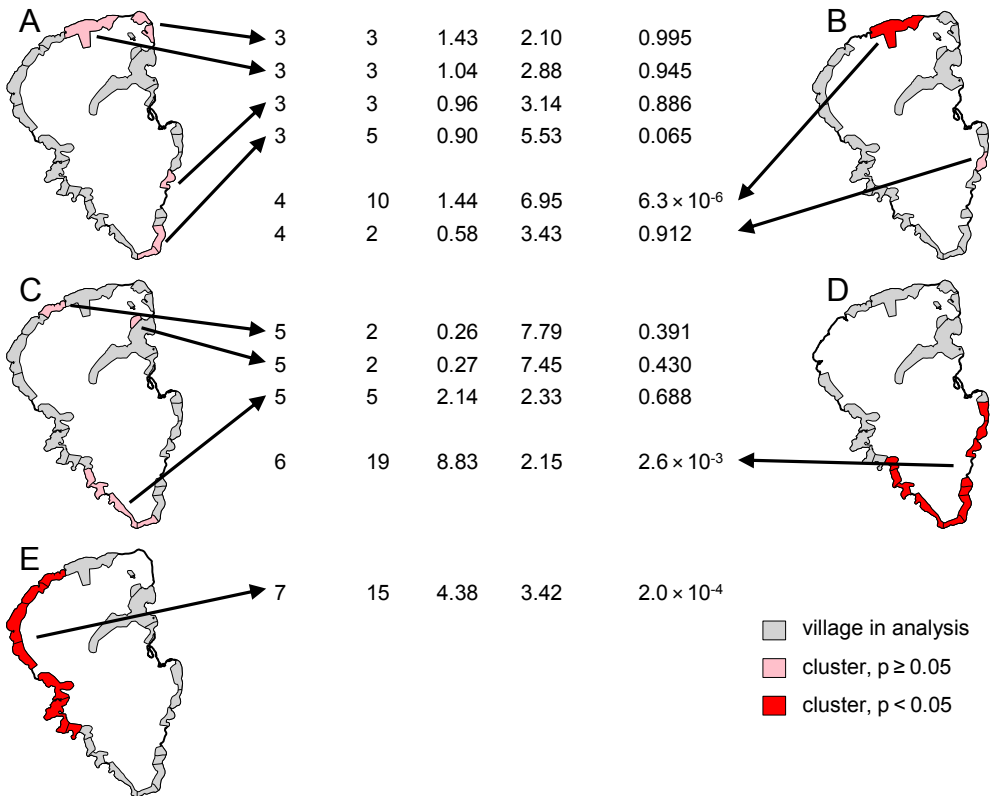

Supplement: S4 Fig — Results of spatial-only discrete Poisson SaTScan analysis for PCR-confirmed prevalent yaws cases identified via active case finding for each survey 3 through 7 (A–E, respectively). Villages that are part of statistically significant spatial clusters are shaded in red and not statistically significant clusters are shaded in pink. Villages are shaded gray if they were part of the analysis in the corresponding survey (but not identified as part of a cluster). Villages are excluded from the map for each survey where the number of individuals screened in that survey in that village is unknown. The arrows point from each cluster to details describing the number of observed yaws cases in that cluster, the number of expected yaws cases, the ratio of observed to expected, and the p-value for that cluster. (PDF) [file pntd.0006840.s008.pdf]
